# Positive Endocervical Margins at Conization: Repeat Conization or Colposcopic Follow-Up? A Retrospective Study

**DOI:** 10.14740/jocmr2171w

**Published:** 2015-05-08

**Authors:** Antonio Chambo Filho, Elediane Garbeloto, Juliana Rodrigues Arrabal Guarconi, Mariana Pereira Partele

**Affiliations:** a Department of Gynecology, Santa Casa de Misericordia Hospital, 143 Dr. Joao dos Santos Neves Street, CEP 29018-180, Vitoria, ES, Brazil

**Keywords:** Conization, Endocervical involvement, Cervix neoplasms

## Abstract

**Background:**

The presence of residual cervical lesions was evaluated in patients submitted to repeat conization due to a finding of positive endocervical margins in a previous loop electrosurgical excision procedure (LEEP) specimen. In addition, the correlation between the presence of a residual lesion and risk factors for cervical cancer, and the use of repeat conization as first-choice treatment were analyzed.

**Methods:**

This retrospective study included 44 patients submitted to repeat cervical conization or total hysterectomy following a finding of affected endocervical margins in LEEP specimens. The risk factors analyzed in relation to the presence of residual lesions were age, smoking, cone depth, glandular involvement and the histopathology findings of cervical intraepithelial neoplasia (CIN) 1, CIN 2 or CIN 3/carcinoma *in situ*. The Chi-square test and the Mann-Whitney *t*-test were used, with significance defined at P < 0.05.

**Results:**

Residual lesions were found in 23/44 patients (52.3%), with 3/23 cases (13.0%) being compatible with invasive squamous cell carcinoma. Of the 23 patients, six (26.1%) were submitted to total hysterectomy, with one case being compatible with a moderately differentiated invasive squamous cell carcinoma. Two patients with a histopathology finding of CIN 3/carcinoma *in situ* in the previous LEEP specimen were diagnosed with invasive squamous cell carcinoma in the repeat conization specimen. Residual lesions were not significantly associated with the risk factors evaluated.

**Conclusions:**

In view of the high frequency of residual disease found when positive endocervical margins were found in LEEP specimens, the indication for repeat cervical conization rather than colposcopic follow-up is viable and justified. Indeed, since the presence of a residual lesion and its progression in the cervical canal are more difficult to screen and control, patients unable to comply with regular colposcopic follow-up could benefit from repeat conization when trying to avoid a potentially negative outcome.

## Introduction

Considered the second most common malignant neoplasm in women worldwide, cervical cancer is the third most common in Brazil after breast and bowel cancer. Its incidence was estimated at 5.7% in Brazil in 2014 [1].

A strong causality relationship has been well established between some types of the human papilloma virus (HPV) and cervical intraepithelial neoplasia (CIN) and cancer. Studies on the HPV types with a high oncogenic potential (HPV types 16 and 18), their viral load and their persistent aggression to the cells have confirmed this. This relationship has also been found in women with compromised immune systems who are infected with HPV, with immune status determining the development and progression of the grades of CIN [2].

The principal methods used to predict the behavior of HPV-induced lesions include cytology, colposcopy and HPV-DNA testing, although the latter is unavailable in many locations [3].

The incidence of cervical intraepithelial lesions has been increasing, particularly in women of reproductive age, a population for whom less aggressive forms of treatment are required [4]. For this reason, cervical conization is now used routinely.

Conization is an extensive form of cervical biopsy in which a cone-shaped wedge of tissue is removed from the cervix for examination. It is recommended when histology findings at biopsy suggest a high-risk lesion; when colposcopy is unsatisfactory, with cytology suggesting pre-invasive cervical disease; when there is a suspicion of micro-invasive/invasive carcinoma or a suspicion of glandular involvement; or when there is a significant discrepancy between cytology and colposcopy [5, 6].

The various methods used for cervical conization include cold knife cone biopsy, laser excisional conization or loop diathermy (loop electrosurgical excision procedure (LEEP)). Of these, LEEP is one of the most commonly used methods for the treatment of cervical intraepithelial lesions, since, in addition to preserving the patient’s reproductive future, cure rates ranging from 60% to 95% have been achieved with this technique [7]. Nevertheless, the endocervical margins are found to be affected in approximately 40% of LEEP specimens and a choice then has to be made between colposcopic follow-up or repeat conization [8].

In fact, residual lesions are found to be present irrespective of the method used for treatment. The therapeutic outcome of LEEP has been shown to be similar to the results achieved with the use of cold knife cone biopsy; however, LEEP is faster, cheaper and involves fewer complications [4].

Wide conization with adequate evaluation of the surgical margins is considered sufficient to treat high-grade lesions. However, the finding of a residual lesion following conization has been significantly associated with margin status, with residual disease being detected in up to 63% of cases with positive endocervical margins [9]. On the other hand, 28% of the women with biopsy specimens showing close margins or thermal artifacts and 8% of those with clear margins also suffer recurrences [6].

Certain factors appear to be associated with a greater risk of residual disease/recurrence. These include age, positive cone margins, the grade of the previous disease and HIV serological status [5]. When these factors are associated, they become the greatest predictors of residual lesions. However, two of these factors have been shown to be more relevant and conflicting: positive margins and extension of glandular involvement in the cone [10].

In the case of positive endocervical margins, repeat conization may benefit some patients, since screening and monitoring of the progression of residual lesions in the cervical canal are difficult tasks. Indeed, patients who are unable to comply with regular colposcopy either because access to healthcare is precarious or for any other reason would be particularly vulnerable to an unfavorable outcome.

## Materials and Methods

This was a retrospective study that analyzed 53 charts of women submitted to repeat conization or to total hysterectomy at the Department of Gynecology, Santa Casa de Misericordia, Vitoria, Espirito Santo, Brazil after having received a histopathology result showing positive endocervical margins in a previous LEEP specimen. The study was approved by the Ethics Committee in Research of the School of Sciences of Santa Casa de Misericordia de Vitoria (CEP/EMESCAM), Espirito Santo, Brazil.

The study encompassed the period between December 2008 and May 2012. HIV-positive patients or those with a history of immunodeficiency, and cases in which data were missing from the medical charts were excluded from the study. A final sample of 44 patients was evaluated.

Histology was performed at the Department of Anatomopathology of the Santa Casa de Misericordia Hospital in Vitoria, Espirito Santo, Brazil.

There was no direct contact with patients while maintaining anonymity. Thus, it was only necessary to query records and confirmation of histopathology in the pathology department of the hospital. The surgical procedures performed are routine service of gynecology and data will be safely stored for 5 years by researchers. Written informed consent was not necessary because patient’s records/information were anonymized and de-identified prior to analysis.

### Statistical analysis

The Statistical Package for the Social Sciences (SPSS), version 16.0 for Windows was used. A descriptive analysis was conducted, using percentages to express categorical variables and means and standard deviations to express the quantitative variables. The Chi-square test was used for categorical variables and the Mann-Whitney *t*-test for the quantitative variables. The significance level adopted was 5%.

The variables analyzed in relation to the risk of finding residual lesions in the sample obtained at repeat conization or total hysterectomy were age, smoking, cone depth, the presence or absence of glandular involvement in the previous cone, and histopathology findings in the lesion (CIN 1, CIN 2 or CIN 3/carcinoma *in situ*).

## Results

Of the 44 patients submitted to repeat cervical conization following a diagnosis of positive endocervical margins in a previous LEEP specimen, 52.3% were found to have a residual lesion. Only two patients also had involvement of the ectocervical margin.

The mean age of the women in the group of patients with a residual lesion was 41.7 years (range 24 - 72 years) compared to 37.1 years (range 26 - 54 years) in the group with no residual lesion (P = 0.204) (Table 1). The mean cone depth at LEEP was 0.55 cm (range 0.3 - 1.2 cm) in the group with a residual lesion compared to 0.84 cm (range 0.3 - 2.0 cm) in the group with no residual lesion (P = 0.065) (Table 1). No statistically significant difference was found between the two groups for either of these variables in relation to the prevalence of a residual lesion.

**Table 1:** Analysis of Age and Cone Depth as Possible Risk Factors for the Presence of Residual Lesions at Repeat Conization

Variables	Groups	Median	Mean	Standard deviation	P value
Women’s age (years)	No residual lesion	38.00	37.10	8.53	0.204
	Residual lesion	42.00	41.70	14.39	
Cone depth (cm)	No residual lesion	0.70	0.84	0.52	0.065
	Residual lesion	0.45	0.55	0.28	

This study found no significant effect of smoking, histological findings (CIN 1, CIN 2 or CIN 3/carcinoma *in situ*) or glandular involvement on the detection rate of a residual lesion. In addition, no cases of CIN 1 were found in the conization samples (Table 2).

**Table 2:** Analysis of the Variables Smoking, Previous Histopathology, and Glandular Involvement in Relation to the Presence of Residual Disease

Variables	No residual lesion		Residual lesion		P value
	N	%	N	%	
Smoking					0.892
Yes	7	41.2	8	36.4	
No	8	47.1	12	54.5	
Former smoker	2	11.7	2	9.1	
Histopathology of repeat cone					1.000
CIN 2	3	14.3	4	17.4	
CIN 3/*in situ* carcinoma	18	85.7	19	82.6	
Glandular involvement					0.802
Yes	12	57.1	14	60.9	
No	9	42.9	9	39.1	
Total		100.0		100.0	-

Of the 23 patients presenting with residual lesions in the repeat cones, six (26.1%) were later submitted to total hysterectomy. In these cases, hysterectomy was performed because there was an invasive lesion or because the woman did not wish to have any more children and/or there was a concomitant benign disease (uterine fibroids) in addition to the finding of positive margins. Of these six cases, one was compatible with a moderately differentiated invasive squamous cell carcinoma, while in two others pathology results were compatible with a squamous cell carcinoma *in situ* and in another with chronic cervicitis/squamous metaplasia. Two patients with a histopathology finding of CIN 3/carcinoma *in situ* in a cone with positive margins were later diagnosed with an invasive squamous cell carcinoma when the repeat conization specimen was evaluated. Overall, three of the 23 patients (13.0%) with positive endocervical margins at the initial conization had a residual lesion compatible with an invasive squamous cell carcinoma. All the patients submitted to total hysterectomy were between 35 and 72 years of age.

## Discussion

Negative margins in a conization sample may not necessarily be indicative of cure, just as positive margins do not necessarily reflect a residual lesion. In fact, incomplete excision of CIN may result in a significant problem when opting for colposcopic follow-up on an outpatient basis, since patients living far from healthcare centers are often unable to comply with scheduled visits, which ultimately facilitates progression of the disease to more advanced stages [11].

Goncalves et al [8] evaluated 35 women submitted to LEEP and reported that, when endocervical curettage was performed immediately following conization in which the specimens had presented positive endocervical margins, residual disease was found in 21.43% of the cases. These values are lower than those found in the present study in which 52.3% of the population sample had a residual lesion. On the other hand, Kietpeerakool et al reported similar findings [12] following repeat LEEP or hysterectomy in 85 patients, with 51.8% having a residual lesion of CIN 2 or 3. Moreover, Kim et al [4] found a high-grade residual lesion in 42.8% of the women submitted to hysterectomy post-LEEP. Felix et al [9], however, reported a residual lesion in 69% of cases when the endocervical margins were affected.

As in the present study, Sommacal et al [7], who prospectively evaluated 16 patients submitted to conization due to a previous diagnosis of CIN 2 or 3, failed to establish any association between smoking or age and the presence of a residual lesion (found in 44.5% of the sample). The mean age of the patients was 34.8 years (range 20 - 60 years), a younger group compared to that in the present study whose mean age was 41.7 years. Ramchandani et al [13] also failed to find any significant correlation between age and a residual lesion when 152 cases of conization were analyzed. The fact that the women in this study were on average older may explain the high rate of residual lesions, since in menopausal patients the squamocolumnar junction is inside the endocervical canal, which increases the risk of disease [12].

Carvalho et al [11] evaluated patients submitted to cone/frozen section examination followed by hysterectomy and reported that the depth of the cone ranged from 1 to 2.5 cm, with 64% of positive margins. Of these, residual disease was found in the uterus in 68.8% of cases. Agreement between frozen section examination and the final result of the cone biopsy was 92%. Although cone depth had no significant effect in the present study, in 52.3% of the cases in which depth was 0.3 - 2.0 cm a residual lesion was present.

In the present study, of the 23 patients with positive margins in the repeat cone biopsy, six (26.1%) were submitted to hysterectomy. In one of these six hysterectomized patients, a moderately differentiated invasive squamous cell carcinoma was found, whereas only a high-grade lesion (CIN 3/carcinoma *in situ*) had been present in the initial and repeat conization specimens. Two cases of invasive squamous cell carcinoma were found in the repeat conization specimens. Therefore, in 13.0% of the patients with positive endocervical margins at conization, findings were compatible with invasive squamous cell carcinoma at the end of treatment.

According to Kietpeerakool et al [12], the only predictor of residual disease is extensive endocervical involvement of the cone margin, with six cases (13.62%) in that study being compatible with invasive carcinoma. Likewise, in a 7-year retrospective study, Chang et al [14] reported that only two out of 33 patients (6.1%) with positive margins in the cones had more advanced residual lesions in the post-conization hysterectomy specimen. Those authors found that the more severe the lesion, the greater the risk of a residual lesion; however, this association was not confirmed in the present study. Other authors have associated the greater risk of residual disease with the severity of the lesion and involvement of the endocervical margin, even in prospective studies [9, 13-15].

In the literature, the presence of a residual lesion has been reported in 8.6-54.8% of cases. This variation may reflect a short time interval between procedures, the state of resection margins in the previous conization or the indications for hysterectomy [4]. Some authors have called attention to the fact that hysterectomy performed within a short period of time (6 weeks) after conization may lead to false-positive results due to inflammatory changes. In the present study, all the patients were submitted to repeat conization following a minimum period of 12 weeks after the previous LEEP. In addition, even in those patients diagnosed with invasive carcinoma at repeat conization, with diagnosis being confirmed later at hysterectomy/lymphadenectomy, there was a 3-month interval between the repeat LEEP and hysterectomy.

The ability of colposcopy to identify areas with more severe lesions has been questioned, and there may be a significant proportion of women with more severe endocervical lesions that remain undetected at colposcopy [4]. Therefore, detecting possible risk factors during post-conization follow-up may help select patients for more extensive monitoring by cytology or colposcopy or even for interventionist management. As a definitive treatment, hysterectomy should be reserved for patients with a report of invasive pathology in the cone, for those with a concomitant uterine morbidity or in the case of patients considered unreliable insofar as compliance with continuous monitoring is concerned.

### Conclusions

Although there was no effect of age, smoking, cone depth, lesion type or glandular involvement on the prevalence of residual lesions, it is well known that expectant management in patients with positive endocervical margins requires strict compliance with follow-up. Otherwise, patients are highly unlikely to receive effective treatment for a cervical lesion.

As with other retrospective studies, this analysis also has its limitations, which include the size of the sample evaluated. These factors may have resulted in the discrepancies found between these results and those of other studies in the literature; however, in view of the high frequency of residual lesions following conization when the endocervical margins are affected, these data are viable and the indication for repeat cervical conization rather than colposcopic follow-up is justified.
